# A Case of Posterior Reversible Encephalopathy Syndrome With Single-Agent Weekly Paclitaxel

**DOI:** 10.7759/cureus.102935

**Published:** 2026-02-03

**Authors:** Mayal Arshad, Syed Umer Ali, Muhammad Sohaib Siddique, Vikram Bansal, Syed Ali Raza, Mateen Akhtar

**Affiliations:** 1 Oncology, Queen's Hospital, London, GBR; 2 Oncology, Sindh Institute of Urology and Transplantation, Karachi, PAK; 3 Accident and Emergency, Din Medical Complex, Burewala, PAK; 4 Oncology, Hull University Teaching Hospitals NHS Trust, Hull, GBR; 5 Medical Oncology, Northwick Park Hospital, London, GBR

**Keywords:** chemotherapy-related toxicity, intraductal carcinoma of breast, paclitaxel, posterior reversible encephalopathy syndrome (pres), systemic anti-cancer therapy (sact)

## Abstract

Posterior reversible encephalopathy syndrome (PRES) is a clinico-radiological condition characterised by acute neurological symptoms and typical magnetic resonance imaging findings. It is commonly associated with hypertension, renal dysfunction, autoimmune conditions, and exposure to certain systemic anti-cancer therapy (SACT) agents. Although PRES has been reported with several chemotherapeutic drugs, its occurrence shortly after the first dose of single-agent weekly paclitaxel is exceptionally rare and not well-documented. We report the case of a 61-year-old female with metastatic breast cancer who developed acute confusion, focal seizures, and a reduced Glasgow Coma Scale (GCS) shortly after receiving her first dose of weekly paclitaxel (80 mg/m²) for visceral crisis. Prior to chemotherapy, she had no history of hypertension, neurological disease, or chronic kidney disease. Following paclitaxel administration, she developed transient hypertension and rapid neurological deterioration. The CT scan was normal. MRI confirmed findings consistent with PRES, showing bilateral parieto-occipital cortical-subcortical signal changes. Paclitaxel was withheld, and she was medically managed (antihypertensive, antiepileptic treatment, and corticosteroids), leading to gradual recovery. This case highlights PRES as a rare but serious potential complication of single-agent paclitaxel therapy. Clinicians should maintain a high index of suspicion for PRES in patients presenting with new-onset neurological symptoms following chemotherapy, as early recognition and prompt management are essential for a favourable outcome.

## Introduction

Paclitaxel belongs to the group of antimicrotubular agents and is formulated from a naturally occurring diterpenoid compound [1]. It promotes the assembly of microtubules whilst simultaneously inhibiting their disassembly. This results in cell cycle arrest at the late G2 phase, therefore preventing cell replication [2].

Paclitaxel was given FDA approval in 1992 for use in ovarian cancers but is also now used as a first-line treatment option in other cancers, including adjuvant treatment of early-stage breast cancer after conclusion of standard chemotherapy with doxorubicin and cyclophosphamide [3].

Paclitaxel is given at a dose of 80 mg/m^2^ once in a cycle of seven days, and this cycle is repeated for a total of 18 cycles [4]. It is associated with quite a few side effects, with the main ones being a hypersensitivity reaction, myelosuppression, peripheral neuropathy, myalgia, hand-foot syndrome, and rare effects on the heart and lungs [4,5]. However, in our case report, we encountered a patient who developed posterior reversible encephalopathy syndrome (PRES). Although commonly associated with other systemic anti-cancer therapy (SACT) agents like tacrolimus, cyclosporine, and cisplatin, this is a side effect rarely associated with paclitaxel shortly after the first dose [6,7].

## Case presentation

This 61-year-old female was admitted with severe hypercalcaemia with an adjusted calcium of 5.05 mmol/L (normal: 2.1 to 2.6 mmol/L). She had a history of being diagnosed with invasive ductal carcinoma (IDC) of the left breast. She underwent a wide local excision and sentinel node biopsy. Histopathological examination confirmed grade 2 IDC, staged as pT1c pN0. The tumour was oestrogen receptor (ER) positive (score 8) and progesterone receptor (PR) positive (score 8), with HER2-negative status. The Nottingham Prognostic Index (NPI) was 4.40, and the Oncotype DX recurrence score was 36. She completed adjuvant chemotherapy consisting of three cycles of the FEC regimen (5-fluorouracil, epirubicin, and cyclophosphamide) and three cycles of docetaxel, followed by adjuvant post-operative radiotherapy to the left breast. Since then, she had been on adjuvant anastrozole and oral ibandronic acid.

However, three years after the adjuvant radiotherapy, she presented with worsening pain in her right pelvis and difficulty in walking. A whole-body bone scan and CT scan showed extensive hepatic metastases with mixed sclerotic and lytic bone lesions. She attended an oncology clinic to discuss treatment, and a blood test done as a baseline showed significant hypercalcaemia, though she has not manifested any related symptoms. She has no other significant comorbidity and no history of hypertension or diabetes.

On day 1 of her admission, her blood pressure was 129/78 mmHg and remained stable for the next 48 hours. Blood results also indicated an acute kidney injury (AKI) score of one with raised urea and creatinine levels. Serum parathyroid, thyroid-stimulating hormone (TSH) and vitamin D levels were within normal limits. Liver function tests were mildly deranged, with elevated alkaline phosphatase levels. During that time, she was treated aggressively with intravenous hydration. On day 3 of her admission, her blood pressure rose to 170/80 mmHg, while her AKI resolved and adjusted calcium levels were reduced to 3.54 mmol/L. Urgent treatment with chemotherapy was planned for the visceral crisis. She received paclitaxel (dose of 80 mg/m^2^ weekly) with denosumab on the same evening to prevent further delay. She was started on paclitaxel single-agent chemotherapy as per the clinician's choice, as the plan was to give cyclin-dependent kinases (CDK) 4/6 inhibitors after that. This was based on the guideline, which is to give chemotherapy first when there is imminent organ failure in metastatic breast cancer [8]. She had a mild reaction to paclitaxel infusion, including a grade 1 rash and itchiness, which resolved with hydrocortisone; the infusion flow rate was then reduced. Post-treatment, her blood pressure increased slightly to 170/90 mmHg, but the National Early Warning System (NEWS) score remained 0. Four hours later, she complained of headaches, followed by the development of newly onset confusion, and in another two hours, she became somnolent with a GCS score falling to 11/15 (E3, V3, M5). Moreover, she developed right-sided focal seizures with repeated jerking movements in her arm and leg. Her blood pressure improved to 130/80 mmHg without intervention. An urgent head CT was performed, which ruled out intracranial pathology.

**Figure 1:**
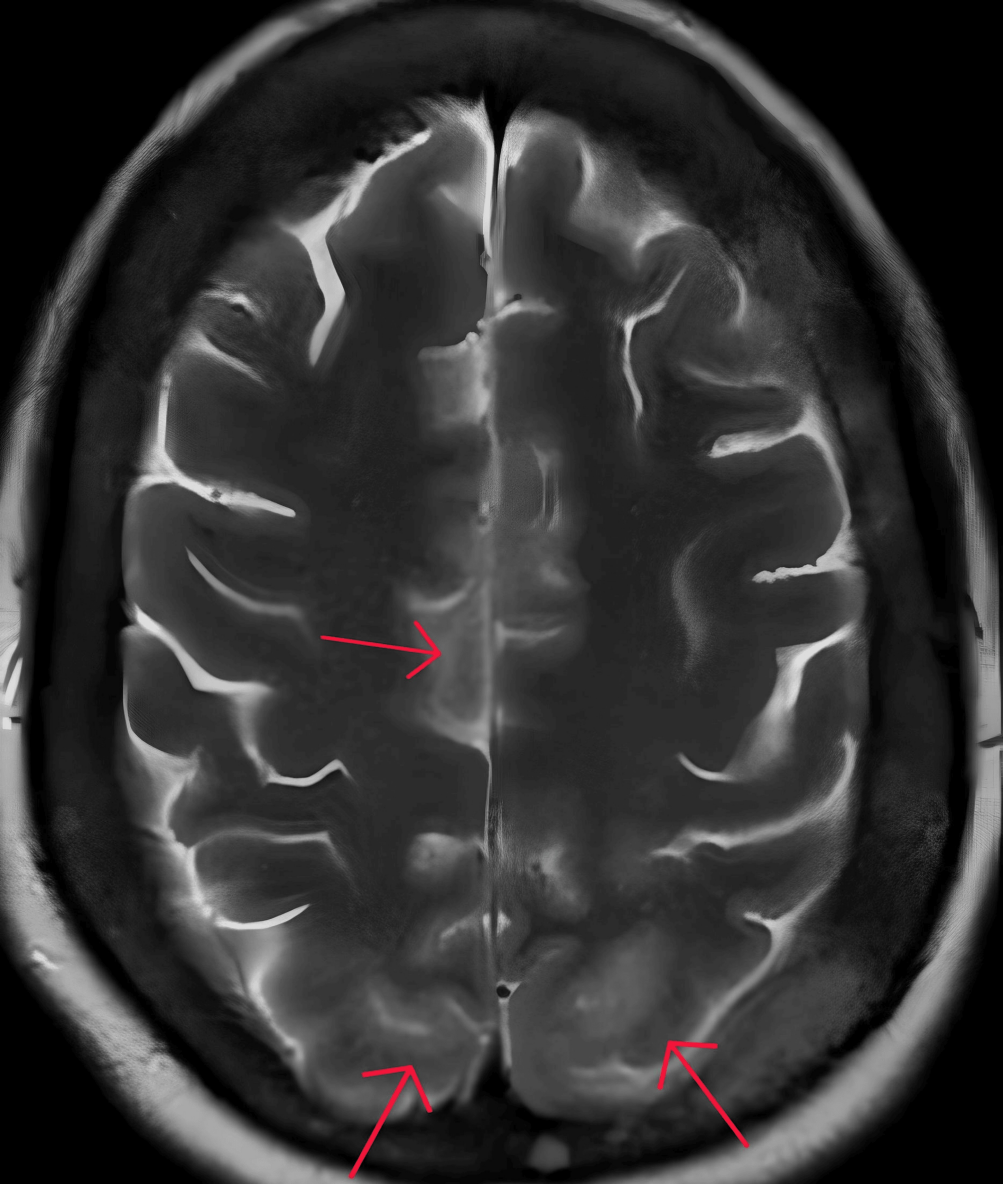
T2-weighted axial MRI slice Multi-focal area of high-signal intensity in the bilateral parieto-occipital lobes

**Figure 2:**
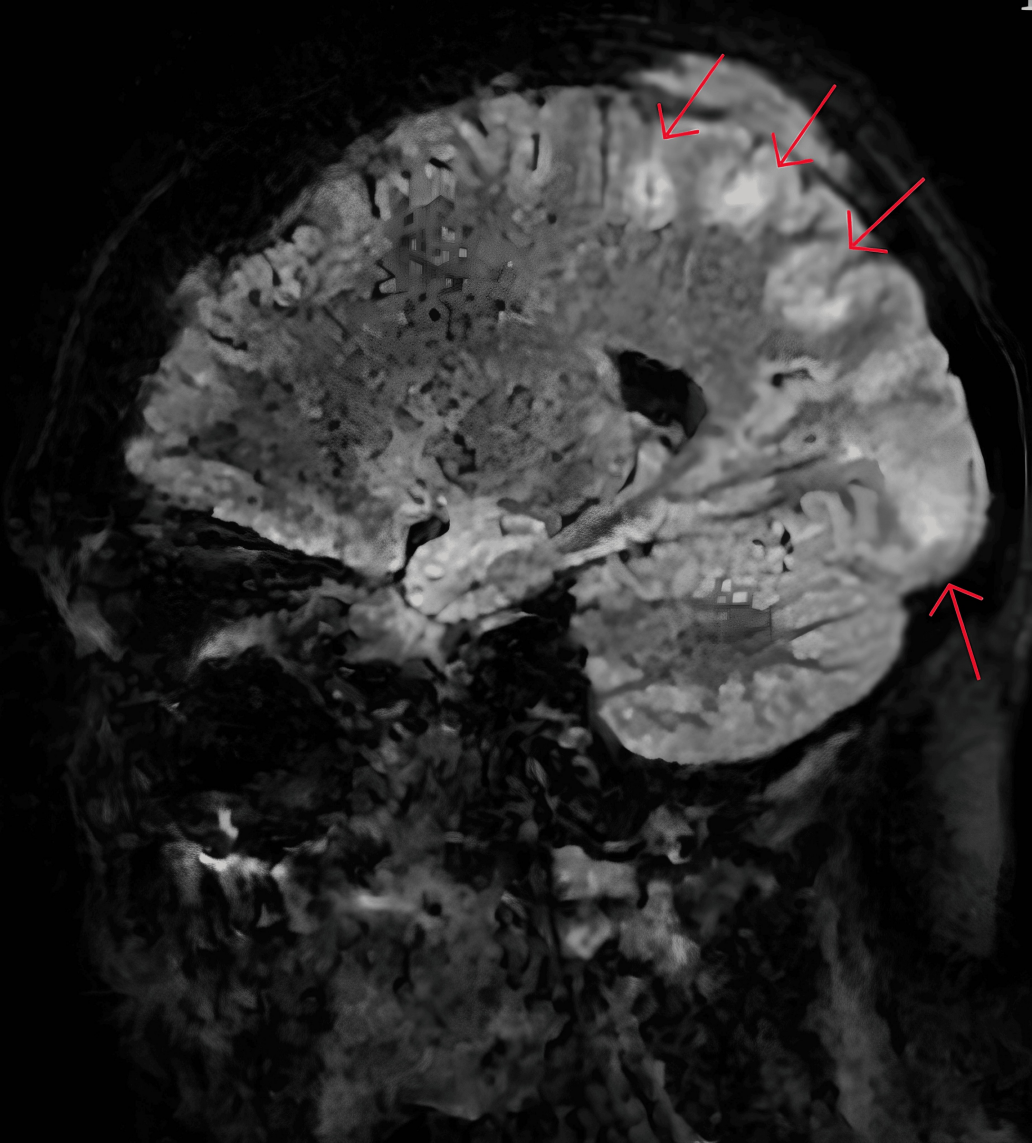
Sagittal FLAIR MRI slice Sub-cortical areas of high-signal intensity FLAIR: fluid-attenuated inversion recovery

**Figure 3:**
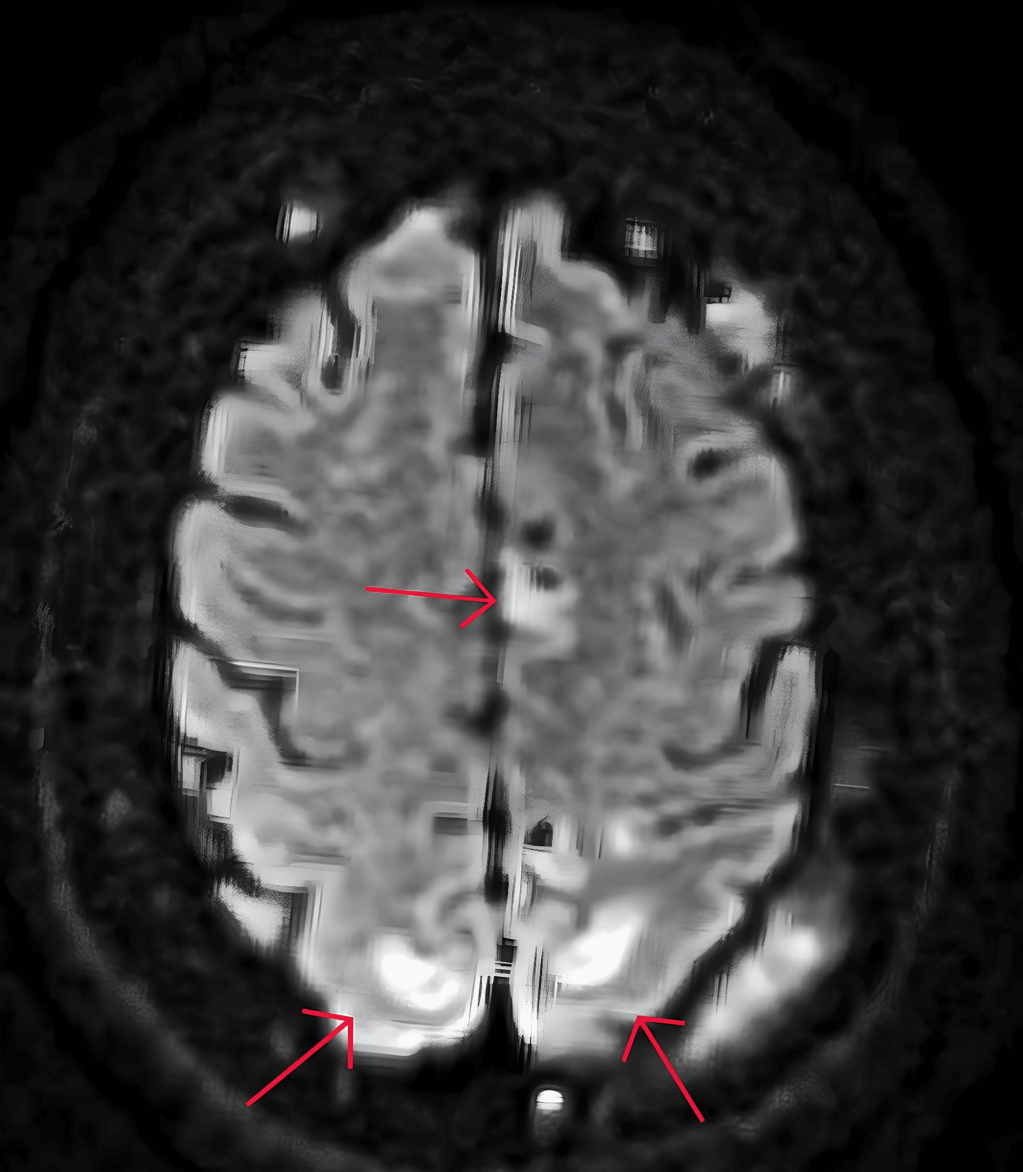
MRI axial DWI slice Multifocal areas of high-signal intensity are seen in the bilateral parieto-occipital lobes DWI: diffusion-weighted imaging

Clinical diagnosis of PRES syndrome was considered based on typical clinical presentation and empirically started on high-dose dexamethasone, keeping a second differential of brain metastasis, awaiting a formal MRI of the head. Lansoprazole cover was given, along with amlodipine and stepping up the dose of levetiracetam. To prevent headaches, six-hourly paracetamol was prescribed. An MRI of the head was conducted within 72 hours, and it confirmed the presence of multifocal areas of cortical-subcortical T2 FLAIR (fluid-attenuated inversion recovery) high signals with associated reduced water diffusion in bilateral parieto-occipital lobes, more pronounced on the left side and also involved the left thalamic pulvinar (Figures 1-3).

She had a gradual recovery and became conscious and alert by day 6 of admission. However, on day 7 of admission, she started developing hallucinations. Her case was discussed with a neurologist, and it was confirmed that the symptoms of hallucinations are unlikely to be related to PRES and are more likely to be related to steroid use. She recovered in 48 hours once we tapered the steroids to a low maintenance level. From day 9 of admission onwards, she was more mobile and started exercises with the physiotherapy team. She had daily blood tests, and her calcium and kidney functions were normalised during this period.

**Figure 4:**
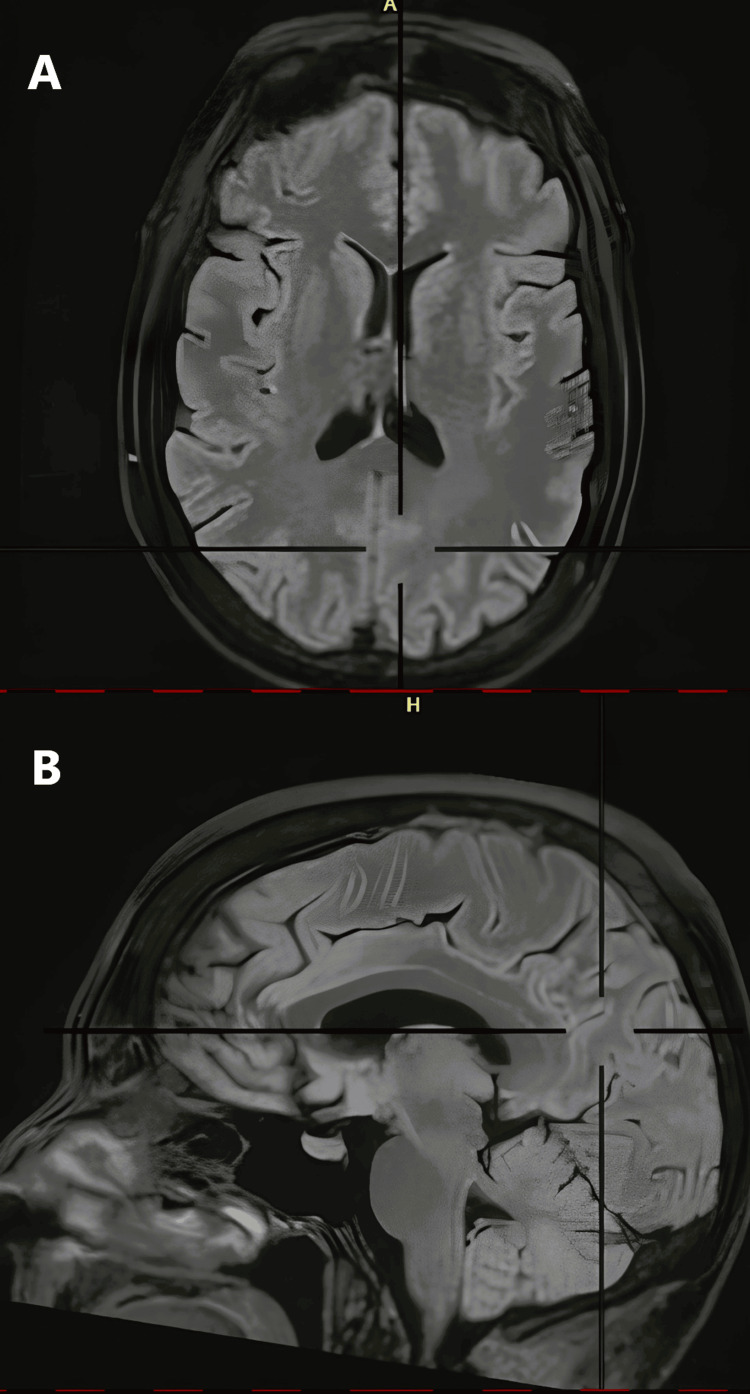
T2 FLAIR (A) axial and (B) sagittal views Resolution of multifocal high-signal intensity areas FLAIR: fluid-attenuated inversion recovery

**Figure 5:**
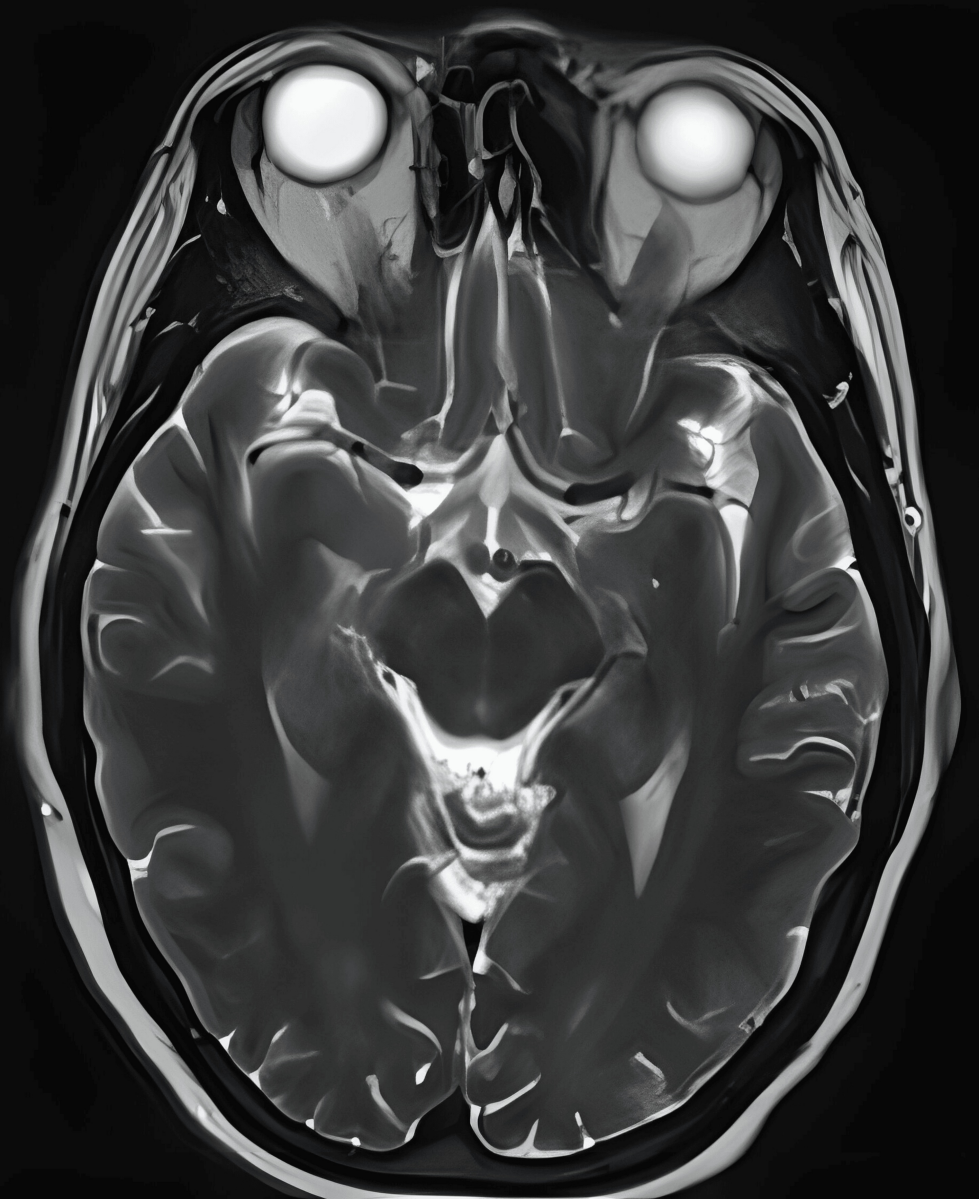
T2-weighted axial slice Resolution of multifocal areas of high intensity

After three months, a follow-up MRI was performed, which showed resolution of the symptoms (Figures 4, 5).

## Discussion

It is believed that PRES, also known as reversible posterior leukoencephalopathy syndrome, occurs due to dysregulation of cerebral autoregulation, which is unable to maintain a constant intracranial blood pressure across a range of systemic blood pressures. The increased hydrostatic blood pressures may break down the blood-brain barrier, causing intravascular fluid to extravasate into the brain tissue, causing brain oedema and associated pressure symptoms [9]. However, other theories exist, and much remains unknown about the complex pathophysiology of PRES. Additionally, unlike its name, it may not even be in the posterior brain, nor may it be reversible.

Although seen in both adult and paediatric populations, the incidence of PRES is still unknown. However, something of note is that it has twice as much likelihood of occurring in females as in males [10].

Amongst the best predictors of PRES in the clinical setting are having an epileptic seizure, having encephalopathy, having exposure to chemotherapy, and having renal failure [11]. Our patient, before receiving paclitaxel, had no CNS or kidney problems. Her blood pressure, already high at 170/80 mmHg, increased further to 170/90 mmHg, followed by a drop in GCS from 15/15 to 11/15.

Previous incidences of PRES due to single-agent paclitaxel have not been reported. In a study involving 579 patients with metastatic breast cancer on paclitaxel, not one developed PRES [12]. In 2015, an incidence of PRES was seen in a 60-year-old patient with lung cancer, six days after she received her third cycle of the carboplatin and paclitaxel regimen [13]. More recently, though, in 2020, a grade 2 invasive ductal breast carcinoma patient on paclitaxel plus trastuzumab treatment developed PRES, after which the treatment regimen was discontinued [14]. PRES has also been reported to occur with the use of atezolizumab [15].

PRES is known to be caused by SACT agents, and unsurprisingly, there has been an increase in the incidence of PRES in the cancer population on SACT agents. With the vast amount of research and development on newer agents being developed, we believe that PRES should be at the top end of the differentials list when it comes to new-onset CNS-related symptoms in the clinical setting, shortly after the administration of SACT agents, be it 'older' ones or new ones. This is because prompt recognition and the initiation of treatment for PRES result in resolution within a couple of weeks.

Although denosumab was initially co-administered with paclitaxel, it is unlikely to be the causative agent, as there is no established association between denosumab and PRES. Its mechanism of action also differs from the pathways typically implicated in PRES, making paclitaxel the more likely trigger.

## Conclusions

This case demonstrates that PRES can occur following single-agent weekly paclitaxel, even in patients without prior neurological disease, chronic hypertension, or significant baseline renal impairment. Importantly, several recognised precipitants of PRES were present, including acute blood pressure elevation, AKI, hypercalcaemia, and aggressive intravenous hydration, highlighting a multifactorial pathophysiology. The patient had documented asymptomatic hypercalcaemia prior to chemotherapy, while the AKI developed after paclitaxel administration, suggesting a temporal relationship between chemotherapy exposure and subsequent physiological derangements. Although paclitaxel is not traditionally recognised as a direct causative agent for PRES, this case supports its role as a potential triggering factor that may lower the threshold for PRES in susceptible patients when combined with concurrent metabolic and haemodynamic stressors. While causality cannot be established from a single case, early recognition, prompt neuroimaging, withdrawal of potential contributing factors, including chemotherapy, and supportive management were associated with clinical improvement. Clinicians should therefore maintain a low threshold for considering PRES in patients who develop acute neurological symptoms following SACT, particularly when multiple risk factors coexist, as timely diagnosis and management remain essential to optimise neurological recovery and minimise the risk of long-term sequelae.
